# Supplementary material for: Anatomy-to-tract mapping infers white matter pathways without diffusion streamline propagation
Source: Nat Commun. 2025 Nov 29;17:36. doi: 10.1038/s41467-025-66615-w (PMC12764483; doi:10.1038/s41467-025-66615-w)
Supplement: Supplementary file 2 — Reporting Summary [file 41467_2025_66615_MOESM2_ESM.pdf]

nature portfolio

Corresponding author(s): Pew-Thian Yap

Last updated by author(s): Nov 1, 2025

Reporting Summary

Nature Portfolio wishes to improve the reproducibility of the work that we publish. This form provides structure for consistency and transparency in reporting. For further information on Nature Portfolio policies, see our Editorial Policies and the Editorial Policy Checklist.

Statistics

For all statistical analyses, confirm that the following items are present in the figure legend, table legend, main text, or Methods section.

n/a	Confirmed	
☐	☒	The exact sample size (*n*) for each experimental group/condition, given as a discrete number and unit of measurement
☐	☒	A statement on whether measurements were taken from distinct samples or whether the same sample was measured repeatedly
☐	☒	The statistical test(s) used AND whether they are one- or two-sided<br>*Only common tests should be described solely by name; describe more complex techniques in the Methods section.*
☒	☐	A description of all covariates tested
☒	☐	A description of any assumptions or corrections, such as tests of normality and adjustment for multiple comparisons
☐	☒	A full description of the statistical parameters including central tendency (e.g. means) or other basic estimates (e.g. regression coefficient) AND variation (e.g. standard deviation) or associated estimates of uncertainty (e.g. confidence intervals)
☒	☐	For null hypothesis testing, the test statistic (e.g. *F*, *t*, *r*) with confidence intervals, effect sizes, degrees of freedom and *P* value noted<br>*Give P values as exact values whenever suitable.*
☒	☐	For Bayesian analysis, information on the choice of priors and Markov chain Monte Carlo settings
☐	☒	For hierarchical and complex designs, identification of the appropriate level for tests and full reporting of outcomes
☒	☐	Estimates of effect sizes (e.g. Cohen's *d*, Pearson's *r*), indicating how they were calculated

Our web collection on statistics for biologists contains articles on many of the points above.

Software and code

Policy information about availability of computer code

Data collection	No data was collected.
Data analysis	The source code and trained models for this study are provided at Zenodo: \url{https://zenodo.org/records/15792527}.<br>Commercial software used: Matlab R2023b<br>Open Source: Python version 3.10.0 and several python libraries (NumPy 1.23.5, PyTorch 2.5.1, Scikit-learn 1.2.1, DiPY 1.8.0, Matplotlib 3.6.3, Nibabel 5.2.0, Scipy 1.9.3), FreeSurfer v7.3.2, MRTrix3 3.0.4, Surflce 13.4, FSL 7.0.6.4, DSI Studio (2023.12.06 "Chen" release), Workbench Command 1.5.0., ScilPy 2.1.0, ATNs 2.5.4

For manuscripts utilizing custom algorithms or software that are central to the research but not yet described in published literature, software must be made available to editors and reviewers. We strongly encourage code deposition in a community repository (e.g. GitHub). See the Nature Portfolio guidelines for submitting code & software for further information.

Data

Policy information about availability of data

All manuscripts must include a data availability statement. This statement should provide the following information, where applicable:

- Accession codes, unique identifiers, or web links for publicly available datasets
- A description of any restrictions on data availability
- For clinical datasets or third party data, please ensure that the statement adheres to our policy

The TractoInferno data used in this study is available at (https://openneuro.org/datasets/ds003900/versions/1.1.1). The SCIL WM atlas used is available at (https://

zenodo.org/records/10103446). Source data are provided with this paper.

Research involving human participants, their data, or biological material

Policy information about studies with human participants or human data. See also policy information about sex, gender (identity/presentation), and sexual orientation and race, ethnicity and racism.

Reporting on sex and gender	Sex and/or gender information was not collected or used for model training or evaluation in this study. The primary objective of this work is methodological development for anatomy-to-tract mapping, and the datasets used were accessed in de-identified form with no available sex or gender metadata. Therefore, no sex-stratified analyses were conducted.
Reporting on race, ethnicity, or other socially relevant groupings	Race, ethnicity, and other socially relevant groupings information were not used in this study.
Population characteristics	Not applicable.
Recruitment	No new participants were recruited.
Ethics oversight	The TractoInferno data is publicly available.

Note that full information on the approval of the study protocol must also be provided in the manuscript.

Field-specific reporting

Please select the one below that is the best fit for your research. If you are not sure, read the appropriate sections before making your selection.

☒ Life sciences ☐ Behavioural & social sciences ☐ Ecological, evolutionary & environmental sciences

For a reference copy of the document with all sections, see nature.com/documents/nr-reporting-summary-flat.pdf

Life sciences study design

All studies must disclose on these points even when the disclosure is negative.

Sample size	We used all available subjects from the TractoInferno dataset (284 subjects total). No formal sample size calculation was performed. Sample sizes were determined by the availability of subjects in the publicly available TractoInferno dataset, which includes 284 subjects and 30 anatomically defined white matter bundles. These numbers are considered sufficient for tractography evaluation and method comparison.
Data exclusions	We did not exclude any of the 284 subjects.
Replication	For the ATM method, reproducibility was ensured for all inference results using the provided model weights (available via the Zenodo link) and code. All ATM predictions can be reproduced exactly using the provided inference scripts. Note that reproducibility statements do not apply to other methods (MRtrix, SCIL atlas warping, TractSeg), as they rely on external pipelines or pre-existing software.
Randomization	Reproducibility was ensured for all inference results using the provided model weights (available via the Zenodo link) and code. All reported predictions can be reproduced using the provided inference scripts.
Blinding	Blinding is not relevant to this study as we are not investigating differences between groups.

Reporting for specific materials, systems and methods

We require information from authors about some types of materials, experimental systems and methods used in many studies. Here, indicate whether each material, system or method listed is relevant to your study. If you are not sure if a list item applies to your research, read the appropriate section before selecting a response.

Materials & experimental systems

n/a	Involved in the study	
☒	☐	Antibodies
☒	☐	Eukaryotic cell lines
☒	☐	Palaeontology and archaeology
☒	☐	Animals and other organisms
☒	☐	Clinical data
☒	☐	Dual use research of concern
☒	☐	Plants

Methods

n/a	Involved in the study	
☒	☐	ChIP-seq
☒	☐	Flow cytometry
☐	☒	MRI-based neuroimaging

Plants

Seed stocks	*Report on the source of all seed stocks or other plant material used. If applicable, state the seed stock centre and catalogue number. If plant specimens were collected from the field, describe the collection location, date and sampling procedures.*
Novel plant genotypes	*Describe the methods by which all novel plant genotypes were produced. This includes those generated by transgenic approaches, gene editing, chemical/radiation-based mutagenesis and hybridization. For transgenic lines, describe the transformation method, the number of independent lines analyzed and the generation upon which experiments were performed. For gene-edited lines, describe the editor used, the endogenous sequence targeted for editing, the targeting guide RNA sequence (if applicable) and how the editor was applied.*
Authentication	*Describe any authentication procedures for each seed stock used or novel genotype generated. Describe any experiments used to assess the effect of a mutation and, where applicable, how potential secondary effects (e.g. second site T-DNA insertions, mosiacism, off-target gene editing) were examined.*

Magnetic resonance imaging

Experimental design

Design type	Not applicable as only T1-weighted and diffusion-weighted images are used.
Design specifications	Not applicable as only T1-weighted and diffusion-weighted images are used.
Behavioral performance measures	Not applicable as only T1-weighted and diffusion-weighted images are used.

Acquisition

Imaging type(s)	Structural T1-weighted and diffusion-weighted imaging.
Field strength	3T
Sequence & imaging parameters	T1w: TR=2400ms, TE=2.14ms, FOV=224x224, voxel size=0.7mm isotropic<br>dMRI: Spin-echo EPI, TR=5520ms, TE=89.5ms, FOV=210x180, voxel size=1.25mm isotropic, b-values=1000,2000,3000 s/mm^2
Area of acquisition	Whole brain

Diffusion MRI ☒ Used ☐ Not used

Parameters: 90 diffusion weighting directions, 3 shells of b = 1000, 2000, 3000 s/mm^2.

Preprocessing

Preprocessing software	The fully pre-processed data provided in TractoInferno was used.
Normalization	The structural data is rigidly aligned to the MNI space and normalized to the intensity range of [0,1] for ATM training.
Normalization template	MNI
Noise and artifact removal	The fully pre-processed data provided by TractoInferno was used.
Volume censoring	No volume censoring was performed.

Statistical modeling & inference

Model type and settings	Anatomy to tract mapping (ATM), which is a deep learning model, was used to produce the main results.
Effect(s) tested	Not applicable.

Specify type of analysis: ☐ Whole brain ☐ ROI-based ☒ Both

Anatomical location(s): Anatomical locations were obtained using the Desikan–Killiany-Tourville (DKT) atlas from Desikan, R. S. et al. An automated labeling system for subdividing the human cerebral cortex on MRI scans into gyral based regions of interest. NeuroImage 31, 968–980 (2006).

Statistic type for inference<br>(See Eklund et al. 2016)	Connectome-level correlation analysis.
Correction	Not applicable as multiple comparisons are not involved.

Models & analysis

n/a	Involved in the study
☐	☒ Functional and/or effective connectivity
☐	☒ Graph analysis
☒	☐ Multivariate modeling or predictive analysis

Functional and/or effective connectivity	Pearson correlation
Graph analysis	Density, characteristic path length, local efficiency, and modularity in the streamline-count weighted graph.
